# Tunable Anomalous Hall Effect in a Kagomé Ferromagnetic Weyl Semimetal

**DOI:** 10.1002/advs.202406882

**Published:** 2024-09-26

**Authors:** Samuel E. Pate, Bin Wang, Yang Zhang, Bing Shen, Enke Liu, Ivar Martin, J. Samuel Jiang, Xiuquan Zhou, Duck Young Chung, Mercouri G. Kanatzidis, Ulrich Welp, Wai‐Kwong Kwok, Zhi‐Li Xiao

**Affiliations:** ^1^ Materials Science Division Argonne National Laboratory Argonne 60439 USA; ^2^ Department of Physics Northern Illinois University DeKalb 60115 USA; ^3^ School of Physics Sun Yat‐sen University Guangzhou 510275 China; ^4^ Department of Physics University of Tennessee Knoxville 37996 USA; ^5^ Min H. Kao Department of Electrical Engineering and Computer Science University of Tennessee Knoxville 37996 USA; ^6^ Institute of Physics Chinese Academy of Sciences Beijing 100190 China; ^7^ Department of Chemistry Northwestern University Evanston 60208 USA

**Keywords:** anomalous Hall effect, frustration, kagomé ferromagnet, weyl semimetal

## Abstract

Emerging from the intricate interplay of topology and magnetism, the giant anomalous Hall effect (AHE) is the most known topological property of the recently discovered kagomé ferromagnetic Weyl semimetal Co_3_Sn_2_S_2_ with the magnetic Co atoms arranged on a kagomé lattice. Here it is reported that the AHE in Co_3_Sn_2_S_2_ can be fine‐tuned by an applied magnetic field orientated within ≈2° of the kagomé plane, while beyond this regime, it stays unchanged. Particularly, it can vanish in magnetic fields parallel to the kagomé plane and even decrease in magnetic fields collinear with the spin direction. This tunable AHE can be attributed to local spin switching enabled by the geometrical frustration of the magnetic kagomé lattice, revealing that spins in a kagomé ferromagnet change their switching behavior as the magnetic field approaches the kagomé plane. These results also suggest a versatile way to tune the properties of a kagomé magnet.

## Graphical abstract

Angle‐dependent Hall resistance measurements show that spins in kagomé ferromagnetic Weyl semimetal Co_3_Sn_2_S_2_ change their switching behavior as the external magnetic field approaches the kagomé plane, enabling the fine tuning of its anomalous Hall effect. These results reveal a remarkable interaction of the external magnetic field and the frustrated magnetic kagomé lattice.

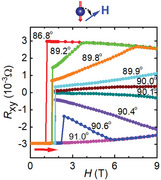

## 1 Introduction

Weyl semimetals are emergent materials hosting relativistic Weyl fermions when inversion symmetry or time‐reversal symmetry is broken.^[^ ^1^, ^2^, ^3^ ^]^ While the inversion symmetry‐broken Weyl semimetals were discovered more than a decade ago and have been extensively studied,^[^ ^1^ ^]^ the time‐reversal symmetry‐broken ones were experimentally confirmed only recently, starting with the ferromagnetic Co_3_Sn_2_S_2_, as revealed by the giant anomalous Hall effects (AHEs)^[^ ^4^, ^5^ ^]^ and characteristic surface Fermi‐arcs and linear bulk band dispersions near the Weyl points.^[^ ^3^, ^6^ ^]^ The interplay of topology and magnetism leads to abundant fascinating phenomena such as anomalous Nernst effect,^[^ ^7^ ^]^ negative magnetoresistance due to a chiral anomaly,^[^ ^4^ ^]^ unusually large magneto‐optic effects,^[^ ^8^ ^]^ and the largest Hall angle reported.^[^ ^9^ ^]^

**Figure 1:**
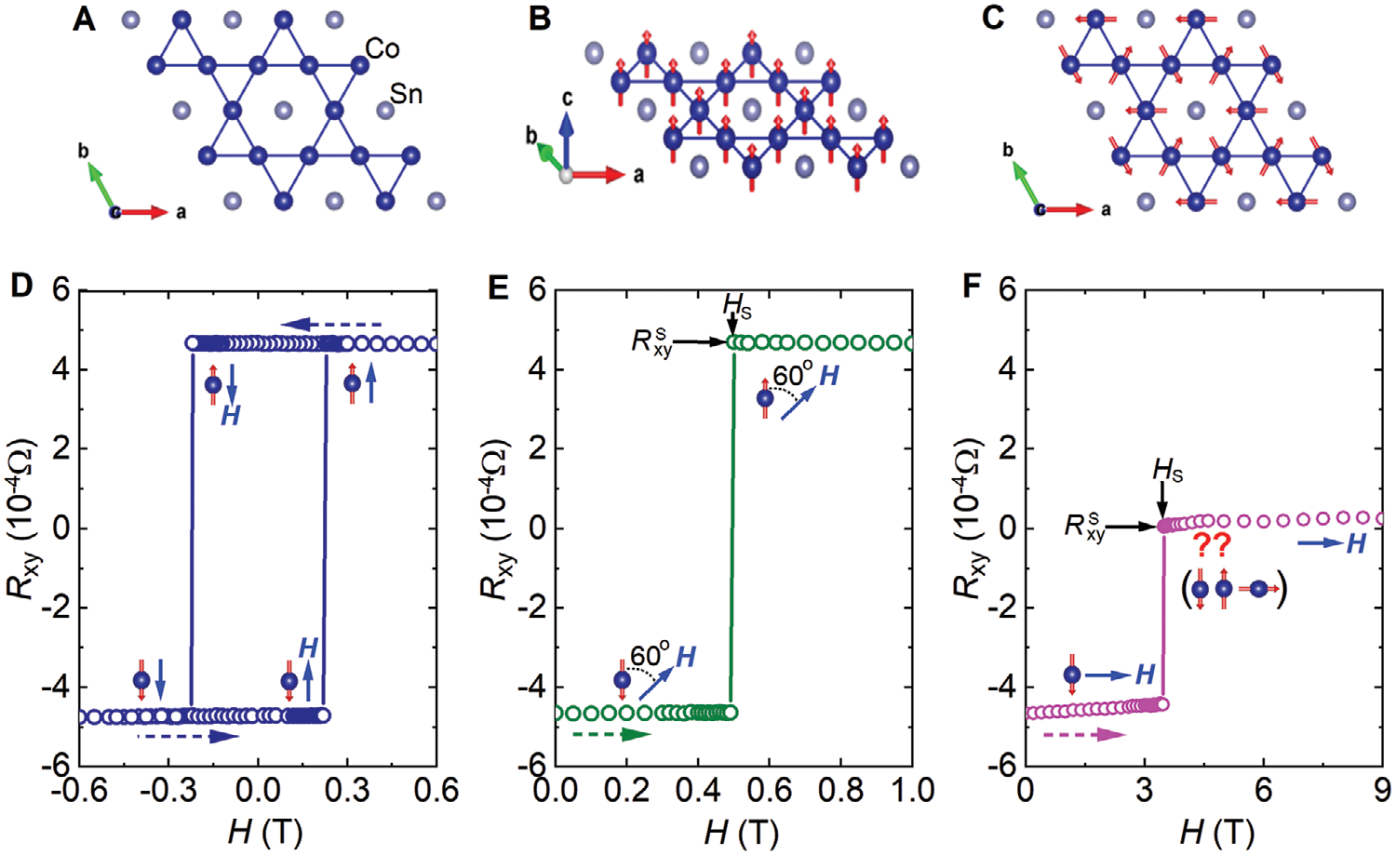
Hall responses of a Co_3_Sn_2_S_2_ crystal. A) Structure of the Co_3_Sn layer containing the Co kagomé lattice. B) Schematic showing one of the FM states with spins along the positive direction of the *c*‐axis. C) Schematic showing one of the AFM states with spins lying in the plane. D) Hysteresis loop of the Hall resistance versus the magnetic field at *H* ∥ *c* (*θ* = 0°). E,F) Hall responses of an FM structure to magnetic fields orientated at *θ* = 60° and 90° (in‐plane), respectively. The FM structure was prepared by sweeping the field up to *H* = −9 T along the *c*‐axis and sweeping back to zero. *θ* is the angle between the positive direction of the *c*‐axis and the field vector pointing in the positive direction of the magnetic field. *H_s_* and $R_{xy}^{S}$ in (E) and (F) are the field and Hall resistance right after the spin switching. More details are presented in Figures S3 and S6 (Supporting Information) and their captions. Data were taken from Sample R1 at *T* = 3 K. The magnetic field rotates in the plane perpendicular to the current direction.

The crystal structure of Co_3_Sn_2_S_2_ comprises Co_3_Sn layers in the *ab* plane separated by SnS_2_ blocks (Figure S1A, Supporting Information),^[^ ^10^ ^]^ with the magnetic Co atoms arranged on a kagomé lattice (**Figure** **1**A; Figure S1B, Supporting Information),^[^ ^11^, ^12^ ^]^ a hallmark of other newly discovered topological kagomé magnets and superconductors with exotic properties.^[^ ^13^ ^]^ Its ferromagnetic (FM) state shows strong anisotropy, with the magnetization easy‐axis along the *c*‐axis (Figure 1B; Figure S1C,D, Supporting Information).^[^ ^14^ ^]^ The combination of the kagomé lattice structure with long‐range out‐of‐plane FM order makes Co_3_Sn_2_S_2_ an excellent candidate for observing exotic topological quantum states such as the quantum anomalous Hall state in the 2D limit.^[^ ^4^, ^15^ ^]^ As revealed in both muon spin‐rotation (µSR)^[^ ^16^ ^]^ and neutron scattering experiments,^[^ ^17^ ^]^ the kagomé lattice could enable the occurrence of frustrated in‐plane anti‐ferromagnetic (AFM) structures (Figure 1C; Figure S1E,F, Supporting Information) competing with the out‐of‐plane FM order at elevated temperatures. While the existence of the in‐plane AFM phases in Co_3_Sn_2_S_2_ has been widely debated,^[^ ^2^, ^11^, ^12^, ^18^, ^19^, ^20^ ^]^ it seems to be the most plausible origin for the observed exchange‐bias effects^[^ ^21^, ^22^ ^]^ that occur typically only in an FM/AFM bilayer structure.

Here we report unusual spin switching behavior enabled by the kagomé lattice in this FM Weyl semimetal. We find that the AHE arising from the FM order in Co_3_Sn_2_S_2_ can be fine‐tuned by applying a magnetic field orientated within ≈2° of the kagomé plane while beyond this regime the behavior follows that of a conventional ferromagnet. Remarkably, the AHE can suddenly vanish as the in‐plane magnetic field reaches a critical value. The amplitude of the AHE can also abruptly decrease at magnetic fields having components even in the same direction as the spins, where no conventional spin flip is expected. The observed AHE behavior can be potentially attributed to local spin switching induced by local lattice instability enabled by the geometrical frustration of the kagomé lattice,^[^ ^18^ ^]^ which converts an ordered FM structure into a mixture of FM domains and in‐plane AFM orders. Our results reveal a remarkable interaction of the external magnetic field and the magnetic kagomé lattice, resulting in distinctive switching behavior of spins in a kagomé magnet. They also shed light on the existence of the highly debated AFM phase in Co_3_Sn_2_S_2_,^[^ ^2^, ^11^, ^12^, ^16^, ^17^, ^18^, ^19^, ^20^ ^]^ which can account for the observed novel phenomena such as bow‐tie‐like hysteresis loops^[^ ^21^ ^]^ and exchange bias effects.^[^ ^21^, ^22^ ^]^

## 2 Results and Discussion

We conducted both transport and magnetization measurements on single crystals grown by flux methods.^[^ ^23^ ^]^ More experimental details including the crystal growth and measurement systems can be found in the Supporting Information. Figure S3 (Supporting Information) and its caption describe the procedures for determining the *ab* plane for precise applied magnetic field orientation. Figure S4 (Supporting Information) shows a typical zero‐field cooling resistance versus temperature curves, revealing a Curie temperature of *T*_c_ ≈ 173 K, consistent with those in the literature.^[^ ^4^, ^5^ ^]^

The FM order in Co_3_Sn_2_S_2_ is believed to be important in the breaking of time‐reversal symmetry, which induces the Weyl states.^[^ ^4^, ^16^, ^17^ ^]^ Current AHE experiments are typically conducted with magnetic fields along the *c*‐axis. Figure 1D and Figure S5A (Supporting Information) present the Hall resistance versus magnetic field (*R*_xy_–*H*) loops of Samples R1 and R2 at *T* = 3 K for *H* ∥ *c*, respectively. They show the typical spin‐flip governed behavior of a ferromagnet. That is, all spins flip when the magnetic field in the opposite direction of the spins reaches the coercive value, resulting in an instantaneous sign change in the *R*_xy_ while retaining its amplitude.^[^ ^24^, ^25^ ^]^ On the other hand, canting the magnetization away from the *c*‐axis is predicted to change the locations of the Weyl nodes, affecting the topological properties such as AHEs and Nernst effects.^[^ ^26^ ^]^ It is also expected to make the FM order unstable.^[^ ^27^ ^]^ Thus, it is interesting to investigate the possible effects of field orientation on topological properties. In fact, recent experiments reveal that magnetic phases in Co_3_Sn_2_S_2_ can depend on the field orientation.^[^ ^28^ ^]^

To further explore the canting effects in the FM state or more generally the response of the FM structure to a tilted external magnetic field, we first create it by applying a field of *H* = −9 T along the *c*‐axis to align all the spins in the same direction. After decreasing the field to zero at which the AHE is observed, we apply the magnetic field at various orientations while recording *R*_xy_ (Figure S6, Supporting Information). As demonstrated in Figure 1E and Figure S5B (Supporting Information) for magnetic fields orientated not too close to the kagomé plane, the FM structure in Co_3_Sn_2_S_2_ behaves the same way as that of a conventional ferromagnet, where flips of all the spins occur and the *R*_xy_ changes sign at the coercive field.^[^ ^24^, ^25^ ^]^ However, an unexpected change occurs in *R*_xy_ when the magnetic field is in the kagomé plane, as presented in Figure 1F and Figure S5C (Supporting Information), which show a sudden change of *R*_xy_ to a value near 0 as the magnetic field increases to a switching field *H*_s_. These results are repeatable (Figure S7A, Supporting Information) in consideration of experimental accuracies such as rotator resolution and reproducibility as well as the nature of the mechanism inducing the sudden change in *R*_xy_, as discussed below. The new state is also stable with field excursion (Figure S7B,C, Supporting Information).

Since the in‐plane saturation field is as high as $H_{ab}^{S}$ = 23 T,^[^ ^14^ ^]^ the sudden occurrence of $R_{xy}^{S}$ ≈ 0 at a much lower field *H*_s_ is not due to the collective flop of the spins from the *c*‐axis to the *ab* plane. While the annihilation of Weyl nodes has only been observed across the Curie temperature,^[^ ^29^ ^]^ it could lead to $R_{xy}^{S}$ ≈ 0 at *H*_s_ if associated with a canted magnetic field.

**Figure 2:**
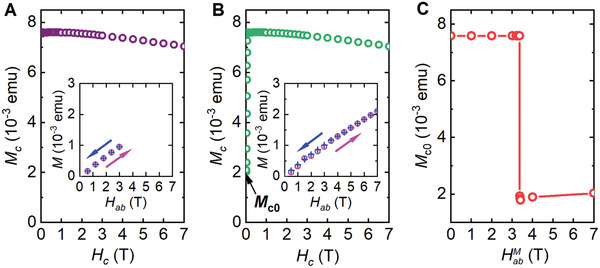
Magnetization of the FM structure being and after driven by an in‐plane field. A,B) *M_c_* versus *H_c_* curves of the FM structure after being driven by an in‐plane field *H_ab_* up to $H_{ab}^{M}$ = 3 T and 7 T, respectively, where *M_c_* is the magnetization measured with the magnetic field *H_c_* aligned along the *c*‐axis. Their insets represent the in‐plane magnetization *M_ab_* for the FM order driven by the in‐plane field *H_ab_*. The FM structure was prepared by sweeping the field up to *H* = 7 T along the *c*‐axis and sweeping it back to zero. Detailed procedures are presented in Figure S8 (Supporting Information) and its caption. C) Relationship between the zero‐field *c*‐axis magnetization *M*_*c*0_ and the $H_{ab}^{M}$. Definition of *M*_*c*0_ is given in (B). Data were taken from Sample M1 at *T* = 3 K.

To clarify these two scenarios, we conducted magnetization measurements on the FM structure driven by an in‐plane field (see Figure S8, Supporting Information for procedures). The insets of **Figure** **2**A,B show that the in‐plane magnetization of the *c*‐axis FM structure of sample M1 increases almost linearly with the in‐plane fields up to *H* = 7 T. We expected to see the in‐plane magnetization vanish if all the spins in the FM structure are perfectly aligned along the *c*‐axis or be the *c*‐axis saturation value if all spins are parallel to the in‐plane field. The smooth increase of the in‐plane magnetization as shown in the insets of Figure 2A,B rules out the possibility of a sudden flop of all the spins from the *c*‐axis to the *ab* plane. On the other hand, it could be induced by spin canting away from the *c*‐axis, which becomes more pronounced at higher in‐plane fields. The main panel of Figure 2A shows the magnetizations measured along the *c*‐axis after the FM structure was driven by in‐plane fields up to *H* = 3 T. It indicates that the FM structure is still intact. In contrast, results in the main panel of Figure 2B reveal that in‐plane fields up to *H* = 7 T can eradicate the FM structure, as shown by the rapid decrease in the *c*‐axis magnetization at zero field, followed by the saturation behavior with increasing field. More measurements following the same procedures with in‐plane field sweeping up to intermediate values, as shown in the main panels of Figure S9A,B (Supporting Information), enable to determine the lowest in‐plane field of 3.37 T for the uniform FM structure to be destroyed. The resemblance of the sudden changes in Figure 2C to those in Figure 1F and Figure S5C (Supporting Information) suggests that the observed vanishing of *R*_xy_ at *H*_s_ is caused by the destruction of uniform FM order, highlighting again the crucial role of FM order on the occurrence of AHE.

**Figure 3:**
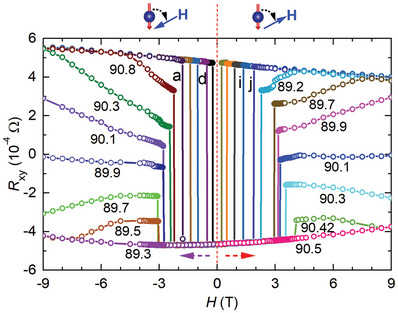
Hall responses to both positive and negative fields at various angles. The FM structure with spins pointing to the negative direction of the *c*‐axis was prepared by sweeping the field up to *H* = −9 T along the *c*‐axis and sweeping back to zero. The red and purple dashed arrows indicate the sweeping directions of the magnetic field for measuring *R*_xy_. Numbers represent the angles at which the *R*_xy_–*H* curves were taken. The angles for the other ten curves (from curve a on the left to curve j on the right) are 92°, 93.8°, 98°, 110°, 180°, 0°, 60°, 75°, 82° and 87.8°. The schematics on top of the main panel show the directions of the spins in the FM structure and the magnetic field as well as the definition of the angle. Data were taken from Sample R1 at *T* = 3 K. The magnetic field rotates in the plane perpendicular to the current direction.

**Figure 4:**
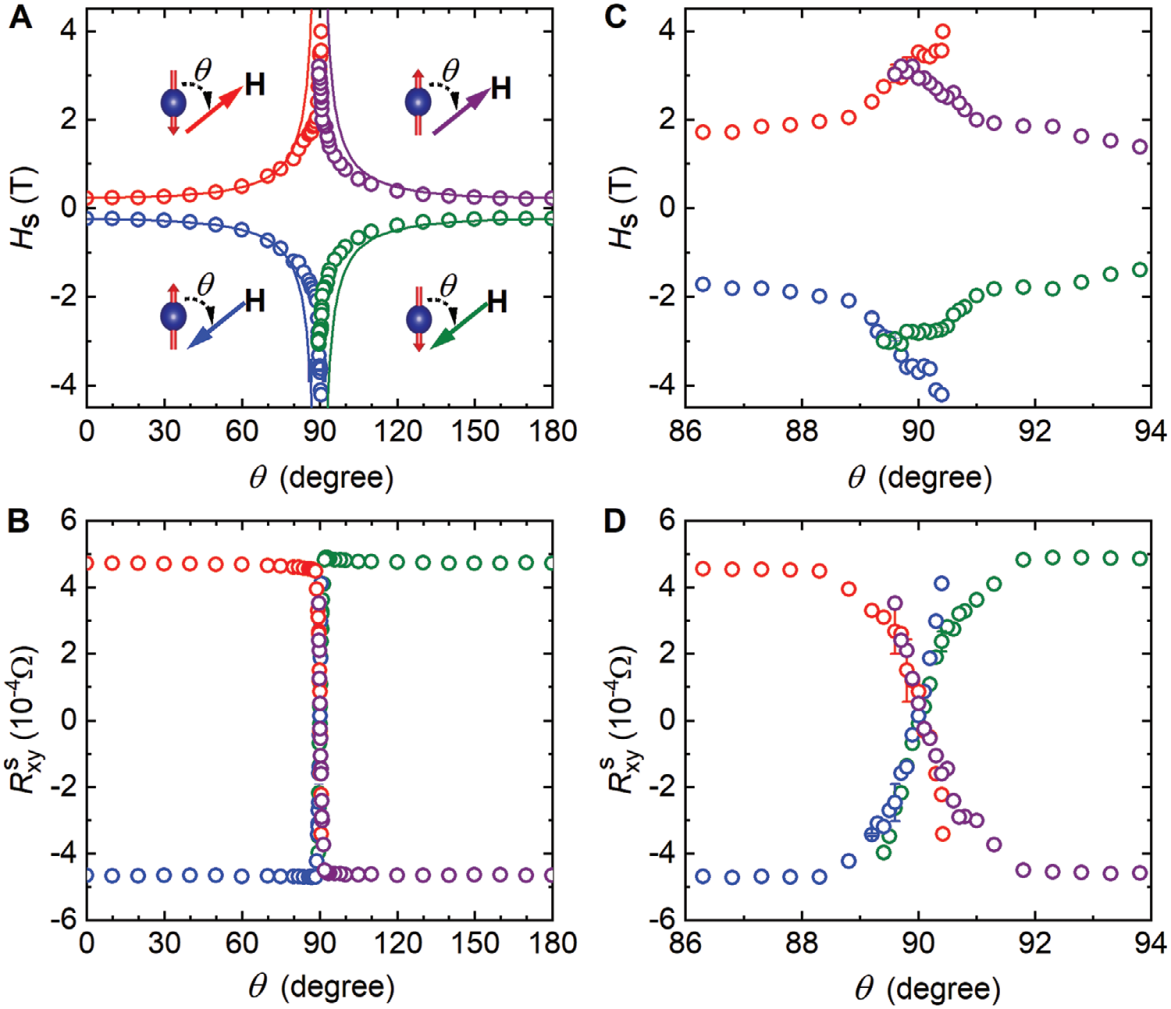
Angle dependence of the field *H*_s_ and Hall resistance $R_{xy}^{S}$ right after the spin switching. A) Angle dependence of *H*_s_. B) Angle dependence of $R_{xy}^{S}$. C,D) Expanded views of (A) and (B) ≈*θ* = 90°, respectively. The schematics in (A) show the directions of the spins in the FM structures and the applied magnetic field as well as the definition of the angle *θ*. Solid lines in (A) represent the Kondorsky relation *H*_s_(*θ*) = *H*_s_(0°)/│cos*θ*│. Data were obtained from measurements similar to those presented in Figure 3.

To gain further insights into the field‐induced suppression of the FM order, we measured *R*_xy_–*H* curves at more angles and in both positive and negative fields to the same FM structure, with emphasis on magnetic fields orientated near the kagomé plane. The results are presented in **Figure** **3**. Consistent with those in Figure 1E and Figure S5B (Supporting Information), *R*_xy_ shows a sudden sign change due to conventional spin‐flips for both directions of the magnetic field as long as they are not aligned too close to the kagomé plane. As presented in **Figure** **4**A, the switching fields can be described with the Kondorsky relation *H*_s_(*θ*) = *H*_s_(0°)/│cos*θ*│,^[^ ^30^, ^31^ ^]^ though deviations can be seen as the field approaches the kagomé plane. Figure 4B shows that the corresponding $R_{xy}^{S}$ stays nearly the same. These results can be accounted for with spin‐flips dominated by the field‐induced nucleation and motion of domain walls that separate regions of opposite uniform magnetizations.^[^ ^32^ ^]^

However, as the field is tilted very close to the kagomé plane, we saw unusual changes in the *R*_xy_. The data in Figure 3 for *θ* = 90.1° in positive fields and 89.9° in negative fields confirm the $R_{xy}^{S}$ ≈ 0 state seen in Figure 1F and Figure S5C (Supporting Information). Besides, we found that $R_{xy}^{S}$ can have other values, as exhibited by the $R_{xy\;\;}^{S}$∼ *θ* curves in Figure 4B,D. That is, the AHE in Co_3_Sn_2_S_2_ can be fine‐tuned with a magnetic field near the kagomé plane. Figure 4 further shows that the FM structures prepared using *H* = ±9 T along both directions of the *c*‐axis behave similarly.

The continuous change of $R_{xy}^{S}$ with angle underlines the mechanism of field‐induced spin instability in destroying the FM order. In fact, abrupt changes in magnetic fields having components even in the same direction of the spins (e.g., at *θ * = 90.3° for positive fields and 89.5° for negative fields in Figure 3) are difficult to reconcile with conventional spin flips. Field‐induced spin instability is also the only reasonable origin for similar changes to an FM structure induced by magnetic fields in opposite directions. As discussed above, spin canting could induce spin instability.^[^ ^27^ ^]^ However, the canting angle *θ*_cs_ = asin(*H*_s_/$H_{ab}^{S}$) < 8° at *H*_s_ is much smaller than the predicted critical angle of 26°.^[^ ^27^ ^]^ Such an instability was expected to induce complete destruction of the FM structure, resulting in $R_{xy}^{S}$ ≈ 0. This is inconsistent with the observed tunability of $R_{xy}^{S}$, which indicate possible partial destruction of the FM structure, i.e., uncompensated FM domains can exist in the states of $R_{xy}^{S}$ ≠ 0.

Very recently, neutron scattering experiments^[^ ^18^ ^]^ revealed that the geometrical frustration in the Co kagomé lattice can cause a local lattice instability, with a symmetry change from hexagonal *R* $\bar{3}$ *m* to distorted monoclinic *Cm*. Density functional theory (DFT) calculations show that this lattice instability causes the local ferromagnetic moments to rotate ≈20°. More importantly, DFT data further show that in the monoclinic lattice, the energies of the in‐plane magnetic states are only slightly higher than those of the out‐of‐plane ones. That is, a local lattice instability can lead to the occurrence of in‐plane magnetic orders, possibly explaining the coexistence of FM and AFM phases^[^ ^16^ ^]^ observed in µSR measurements or the glass phases^[^ ^21^ ^]^ proposed to understand the bow‐tie‐like hysteresis loops occurring in the magnetic field aligned along the *c*‐axis as the temperature is swept up to the Curie temperature^[^ ^21^ ^]^ or at low temperatures when the magnetic field is rotated from the *c*‐axis toward the *ab* plane.^[^ ^28^ ^]^

While the local lattice instability revealed in neutron scattering^[^ ^18^ ^]^ is temperature‐driven, we hypothesize that it can also be induced by a magnetic field, enabling instability at lower temperatures. The accompanying local ferromagnetic instability could result in the observed unusual *R*_xy_ behavior. The application of a magnetic field of *H* = ±9 T along the *c‐*axis stabilizes a perfect FM structure. The interaction of the Co magnetic moment with a magnetic field in or near the plane distorts the Co kagomé lattice and thus could cause a local lattice instability at *H*_s_. The associated local ferromagnetic instability then transforms the ordered FM structure into a mixture of FM domains with local lattices of *R* $\bar{3}$ *m* and *Cm* symmetries as well as in‐plane magnetic orders, probably AFM structures with a local lattice of *Cm* symmetry. The competition of the FM domains and AFM domains of various volumes and directions determines the value of the $R_{xy}^{S}$, leading to its tunability. In fact, the field dependence of *R*_xy_ in Figure 3 with fields *H* > *H*_s_ on both sides of the plane, i.e., at *θ* ≠ 90°, can only be accounted for with such an instability while excluding other mechanisms such as spin‐canting induced instability which leads to the complete destruction of the FM order, i.e., *R*_xy_ = 0.^[^ ^27^ ^]^ Figure 4 also shows that the *H*_s_ ∼ *θ* curves for the same magnetic torque directions reflect each other while exhibiting significant deviations for opposite magnetic torque directions, as expected from a field‐induced local lattice instability since the exchange interactions from the neighbors of a spin in a kagome lattice are not antisymmetric with respect to the magnetic torques induced by opposite magnetic fields (except when the torques are perpendicular to the reflection axes, which is rarely achievable in experiments). On the other hand, the $R_{xy}^{S}$ ∼ *θ* curves for the FM structures of the same magnetization direction in Figure 4B,D nearly overlap each other with a rotation of 180°, demonstrating again that AHEs are governed by the FM orders. The repeatable formation of a perfect FM order by applying a field of *H* = ±9 T along the *c*‐axis and the field‐driven recovery of the FM order after the instability exhibited in Figure 3 at *H* > *H*_s_ and *θ* ≠ 90° imply that an external magnetic field can also clean up the distorted local lattice of *Cm* symmetry, restoring *R* $\bar{3}$ *m* symmetry of the lattice. In fact, the regime (< 2°) near the kagomé plane where *R*_xy_ can be tuned seems to be limited by the saturation field $H_{c}^{S}$ in the *c*‐axis direction. That is, the field‐induced local FM instability may still occur outside this angle regime but the accompanying mixed phases will be cured since the *c*‐axis component (*H*_s_ *sin*2°) of the field is larger than $H_{c}^{S}$ (≈0.1 T).^[^ ^14^ ^]^ While verifications using techniques such as neutron scattering^[^ ^18^ ^]^ are needed, the creation and removal of distorted local lattices by applying a magnetic field suggested by our results constitute a versatile way to tune the properties of a kagomé magnet.

While data presented in Figures 1, 2, 3, 4 were all taken at *T* = 3 K, we did carry out experiments at high temperatures as long as an FM structure at zero field can be prepared. Figure S10 (Supporting Information) shows results obtained at *T* = 80 K, unveiling the same unusual *R*_xy_ behaviors as those at *T* = 3K (Figure 3). We also present in Figure S11 (Supporting Information) the temperature dependence of the switching field *H*_s_ for *H* ∥ *ab* and *H* ∥ *c* as well as their ratios. Clearly, *H*_s_ decreases with increasing temperature for both *H* ∥ *ab* and *H* ∥ *c* while we could obtain $H_{s}^{ab}$ (= 0.46T) in the *ab* plane up to *T* = 150 K at which $H_{s}^{c}$ (= 4 mT) for *H* ∥ *c* is close to zero. Notably, the ratio $H_{s}^{ab}/H_{s}^{c}$ changing from 24 at *T* = 130 to 115 at *T* = 150 K increases much faster with temperature at *T* > 130 K, where FM/AFM coexistence^[^ ^16^ ^]^ and bow‐tie‐like hysteresis loops at *H* ∥ *c* ^[^ ^21^ ^]^ were reported and accounted for with local FM instability.^[^ ^18^ ^]^ In fact, the fast increase in the ratio can also be explained by local FM instability, because the $H_{s}^{c}$ at *H* ∥ *c* decreases faster with increasing temperature due to temperature‐enhanced local FM instability while the $H_{s}^{ab}$ for *H* ∥ *ab* dominated by the field‐induced local FM instability is less sensitive to the temperature.

**Figure 5:**
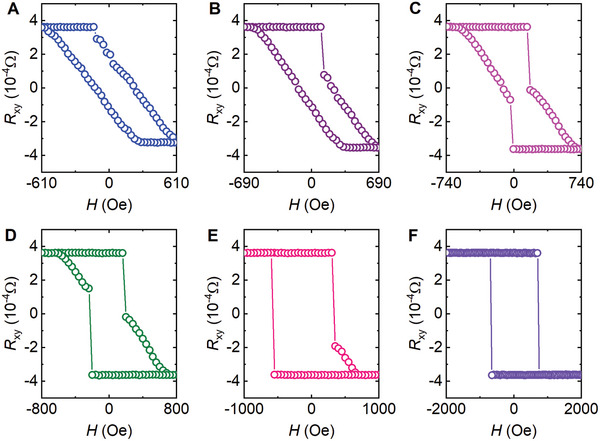
Bow‐tie‐like Hall resistance hysteresis loops. A–F) Results with magnetic fields sweeping up to *H* = 610 Oe, 690 Oe, 740 Oe, 800 Oe, 1 kOe, and 2 kOe, respectively. The *R*_xy_–*H* curves were taken with magnetic fields along the *c*‐axis (*θ* = 180°) after driving the FM structure with an in‐plane field up to *H_ab_* = 3.5 T. The FM structure was prepared by sweeping the field up to *H* = −9 T along the *c*‐axis and sweeping back to zero. Detailed procedures are presented in Figure S12 (Supporting Information) and its caption. Data was taken from Sample R3 at *T* = 3 K.

In this high‐temperature regime we also observed bow‐tie‐like hysteresis loops at *H* ∥ *c*, as shown in Figure S11 (Supporting Information), indicating that they arise from the mixed phases produced by the temperature‐driven local FM instability. While the local FM instability for *H* ∥ *ab* is driven by the applied magnetic field instead of temperature, it should result in a similar mixture of various magnetic phases. Thus, we expect to see bow‐tie‐like hysteresis loops in the FM structure after the occurrence of the field‐driven local FM instability. As presented in **Figure** **5** for *T* = 3 K and Figure S13 (Supporting Information) for *T* = 100 K, the *R*_xy_ hysteresis loops taken at *H* ∥ *c* after the FM structure had been driven by an in‐plane field *H_ab_* > *H*_s_ indeed show bow‐tie‐like features (see Figure S12, Supporting Information for measurement procedures). These results reveal that bow‐tie‐like features of the *R*_xy_ hysteresis loop are hallmarks of a local FM instability driven either by temperature or an applied magnetic field. They provide strong evidence for the existence of the hypothesized field‐induced local FM instability and also support the understanding^[^ ^18^ ^]^ of the origin of the FM/AFM coexistence^[^ ^16^ ^]^ and the bow‐tie‐like hysteresis loops at *H* ∥ *c* ^[^ ^21^ ^]^ at elevated temperatures.

## 3 Conclusion 

We conducted angle‐dependent Hall resistance measurements on kagomé ferromagnetic Weyl semimetal Co_3_Sn_2_S_2_, which reveal that the spins change their switching behavior as the external magnetic field approaches the kagomé plane. Away from the kagomé plane, the spins driven by a magnetic field with a component in the opposite direction flip globally, resulting in a sudden sign change in the anomalous Hall effect (AHE) while retaining its amplitude. When the applied magnetic field is orientated within ≈2° of the kagomé plane local spin switching occurs, yielding fine‐tuning of the AHE. Particularly, it can vanish in magnetic fields parallel to the kagomé plane and even decrease in magnetic fields collinear with the spin direction. We attribute the observed tunable AHE to the occurrence of local ferromagnetic instability caused by distorted local lattices induced by the applied magnetic field due to its interaction with the frustrated magnetic kagomé lattice.

## 4 Experimental Section

### Crystal Growth and Characterizations

Single crystals of Co_3_Sn_2_S_2_ were grown by flux method with tin as the flux. Stoichiometrical metal powders of Co (99.999%), Sn (99.999%), and S (99.95%) were mixed thoroughly in an alumina crucible and then sealed into an evacuated quartz tube. The mixture was first heated to 1050 °C and kept at that temperature for 10 h. After that, the mixture was slowly cooled down to 740 °C over 5 days, and single crystals were separated from the Sn flux by centrifugating. The inset of Figure S2 (Supporting Information) shows an image of a millimeter‐sized crystal. The crystal structure was characterized by an X‐ray diffractometer (Panalytical Empyrean), with a typical XRD pattern presented in Figure S2 (Supporting Information). The stoichiometry of the components was confirmed by using the Energy Dispersive Spectrometer (BRUKER EDS) equipped with a scanning electron microscope (EVO MA10).

### Transport Measurements

Resistance measurements were conducted on four crystals (hereby named Samples R1‐R4) using the Electrical Transport Option (ETO) of Quantum Design PPMS, which showed consistent results. Electrical leads were gold wires glued to the crystal using silver epoxy H20E. A low‐frequency (21.36 Hz) ac current of 1 mA was used and flows in the *ab* plane. Angular dependencies of the resistance were obtained by placing the sample on a precision, stepper‐controlled rotator with an angular resolution of 0.05°. The magnetic field could rotate in a plane perpendicular to the current (Samples R1, R3, and R4) or in the plane determined by the *c*‐axis and the current direction (Sample R2). The magnetic field orientation is represented by *θ*, which is defined as the angle between the positive direction of the *c*‐axis and the magnetic field vector pointing in the positive direction of the magnetic field, as shown in Figure 4A. More information on transport measurement procedures can be found in Figures S3 and S6 (Supporting Information) as well as their captions.

### Magnetization Measurements

Magnetization measurements were carried out in a Quantum Design MPMS3 on two crystals (hereby named Samples M1 and M2). The change of the magnetic field orientation relative to the c‐axis of the crystal was realized by placing the sample on a horizontal rotator (Model 301), which had an angular step size < 0.1°. The definition of θ was the same as that in transport measurements, i.e., θ = 0° (180°) for the magnetic field along the positive (negative) direction of the c‐axis and θ = 90° for the field being parallel to the ab plane. More information on magnetization measurement procedures is presented in Figure S8 (Supporting Information) and its caption.

## Conflict Of Interest

The authors declare no conflict of interest.

## Supporting information



Supporting Information

## Data Availability

The data that support the findings of this study are available from the corresponding author upon reasonable request.
