# Supplementary material for: Tunable Anomalous Hall Effect in a Kagomé Ferromagnetic Weyl Semimetal
Source: Adv Sci (Weinh). 2024 Sep 26;11(43):2406882. doi: 10.1002/advs.202406882 (PMC11578337; doi:10.1002/advs.202406882)
Supplement: Supplementary file 1 — Supporting Information [file ADVS-11-2406882-s001.pdf]

ADVANCED SCIENCE
Open Access

Supporting Information

for *Adv. Sci.*, DOI 10.1002/advs.202406882

Tunable Anomalous Hall Effect in a Kagomé Ferromagnetic Weyl Semimetal

*Samuel E. Pate, Bin Wang, Yang Zhang, Bing Shen, Enke Liu, Ivar Martin, J. Samuel Jiang, Xiuquan Zhou, Duck Young Chung, Mercouri G. Kanatzidis, Ulrich Welp, Wai-Kwong Kwok and Zhi-Li Xiao**

Supporting Information

Tunale Anomalous Hall Effect in a Kagomé Ferromagnetic Weyl Semimetal

*Samuel E. Pate, Bin Wang, Yang Zhang, Bing Shen, Enke Liu, Ivar Martin, J. Samuel Jiang, Xiuquan Zhou, Duck Young Chung, Mercouri G. Kanatzidis, Ulrich Welp, Wai-Kwong Kwok, and Zhi-Li Xiao**

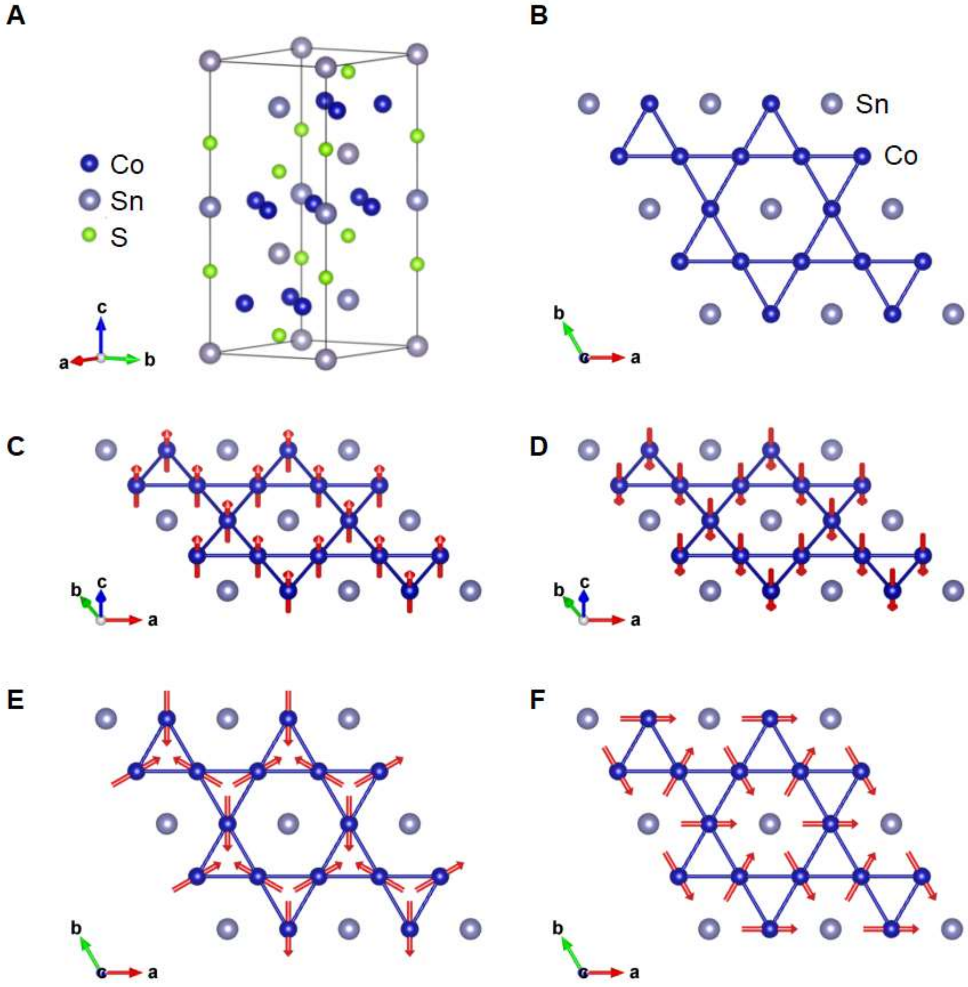

Figure S1. Crystal and magnetic structures of $Co_3Sn_2S_2$. (**A**) Unit cell. (**B**) Top view of the Co_3Sn plane. (**C**) and (**D**) Ferromagnetic orders with spins along the positive and negative directions of the *c*-axis, respectively. (**E**) and (**F**) Anti-ferromagnetic structures with $R\overline{3}m'$ (E) and $R\overline{3}m$ (F) symmetries, respectively. Images were produced using VESTA opensource crystal modeling software.[11] Lattice parameters were taken from mp-19807: $Co_3(SnS)_2$ (Trigonal, $R\overline{3}m$, 166) (materialsproject.org).

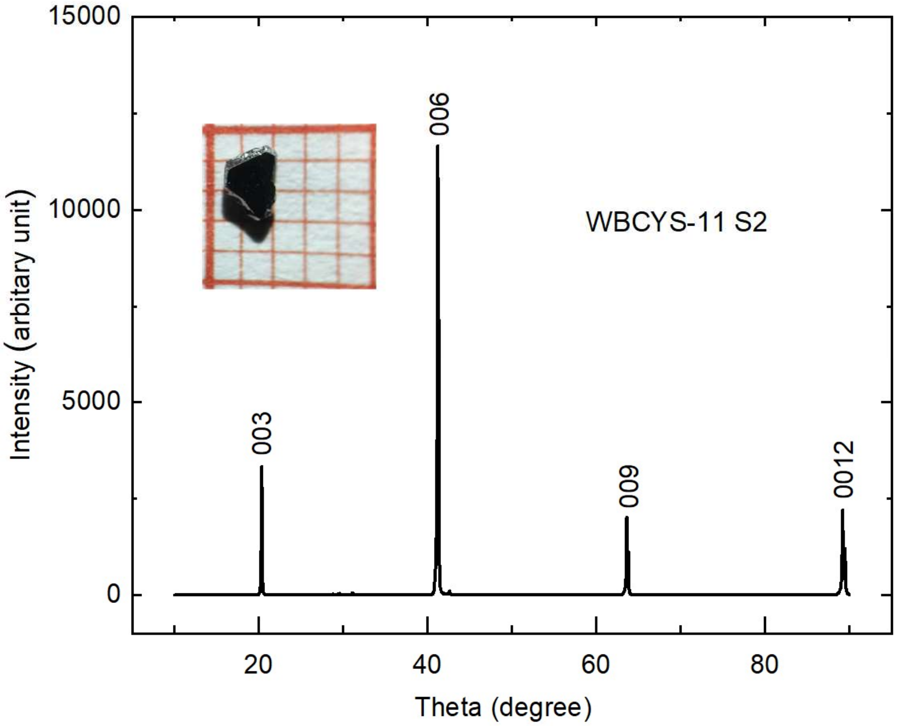

Figure S2. X-ray diffraction pattern of a $Co_3Sn_2S_2$ crystal. The peaks can be indexed by the indices of (00*l*) lattice planes. Inset presents a photo of a typical single crystal on a 1 mm grid paper.

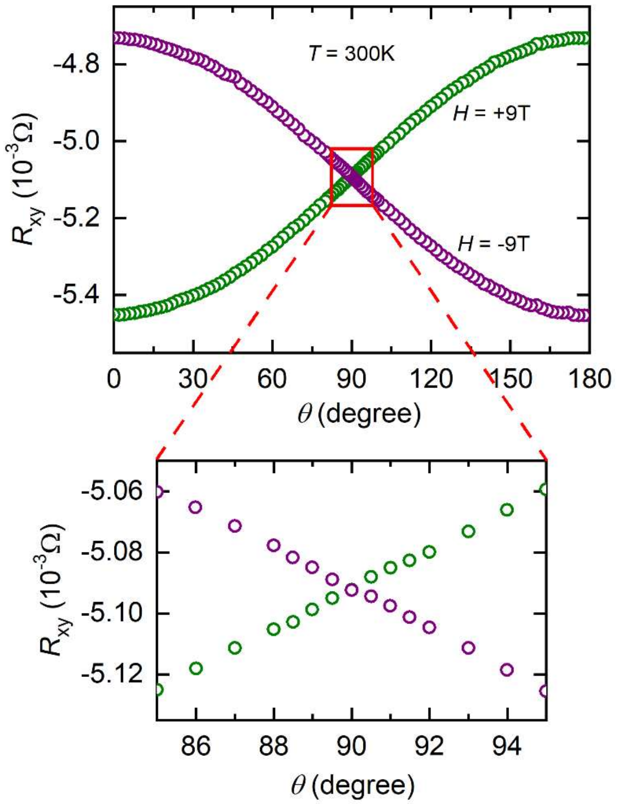

Figure S3. Determination of the kagomé (*ab*) plane. Hall resistance versus angle ($R_{xy} \sim \theta$) curves were taken at H = +9 T and -9 T. The angle at which the two curves cross each other is defined as $\theta = 90°$, corresponding to the kagomé (*ab*) plane. Data were taken from Sample R2 at T = 300 K.

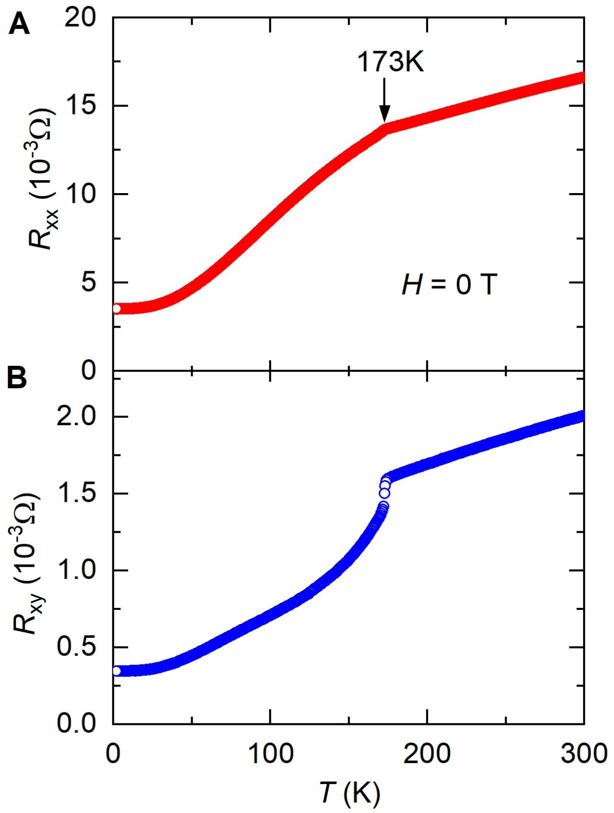

Figure S4. Cooling resistance curves of a $Co_3Sn_2S_2$ crystal. (**A**) and (**B**) present the longitudinal resistance R_{xx} and the Hall resistance R_{xy}, respectively. Data were taken from Sample R2 at zero field. The Curie temperature of $T_c \approx 173$ K is indicated in (A).

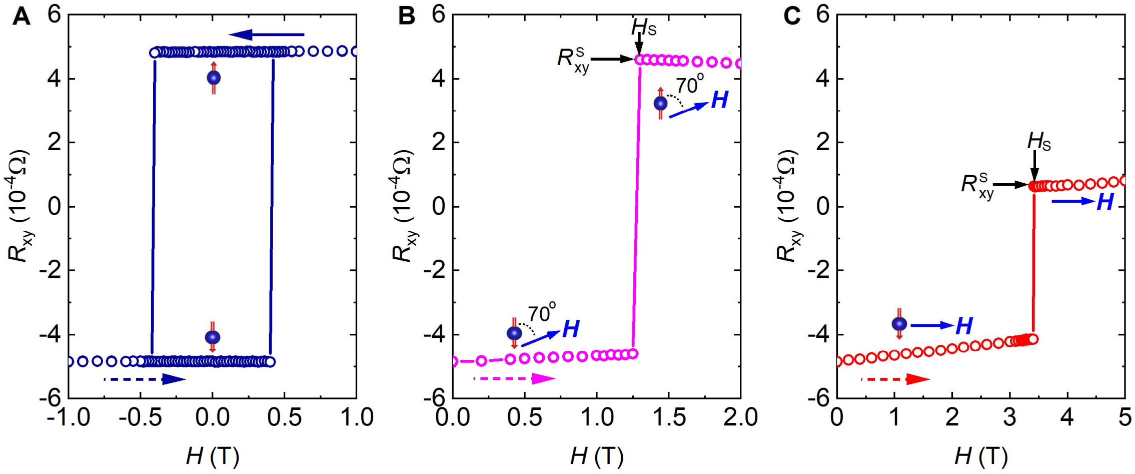

Figure S5. Hall responses of Sample R2. (**A**) Hysteresis loop of the Hall resistance versus the magnetic field at *H*//*c*. The magnetic field was swept up to $H = \pm 9$ T. (**B**) and (**C**) Hall responses of an FM order to magnetic fields orientated at $\theta = 70°$ and 90° (in-plane), respectively. The FM order was prepared by sweeping the field up to $H = -9$T along the *c*-axis and sweeping back to zero. The magnetic field rotates in the plane defined by the current direction and the *c*-axis, with θ being the angle between the positive direction of the *c*-axis and the field vector pointing in the positive direction of the magnetic field (Figure 4A). $\boldsymbol{H_s}$ and $\boldsymbol{R^S_{xy}}$ in (B) and (C) are the field and Hall resistance right after the spin switching. Data was taken at $T = 3$ K.

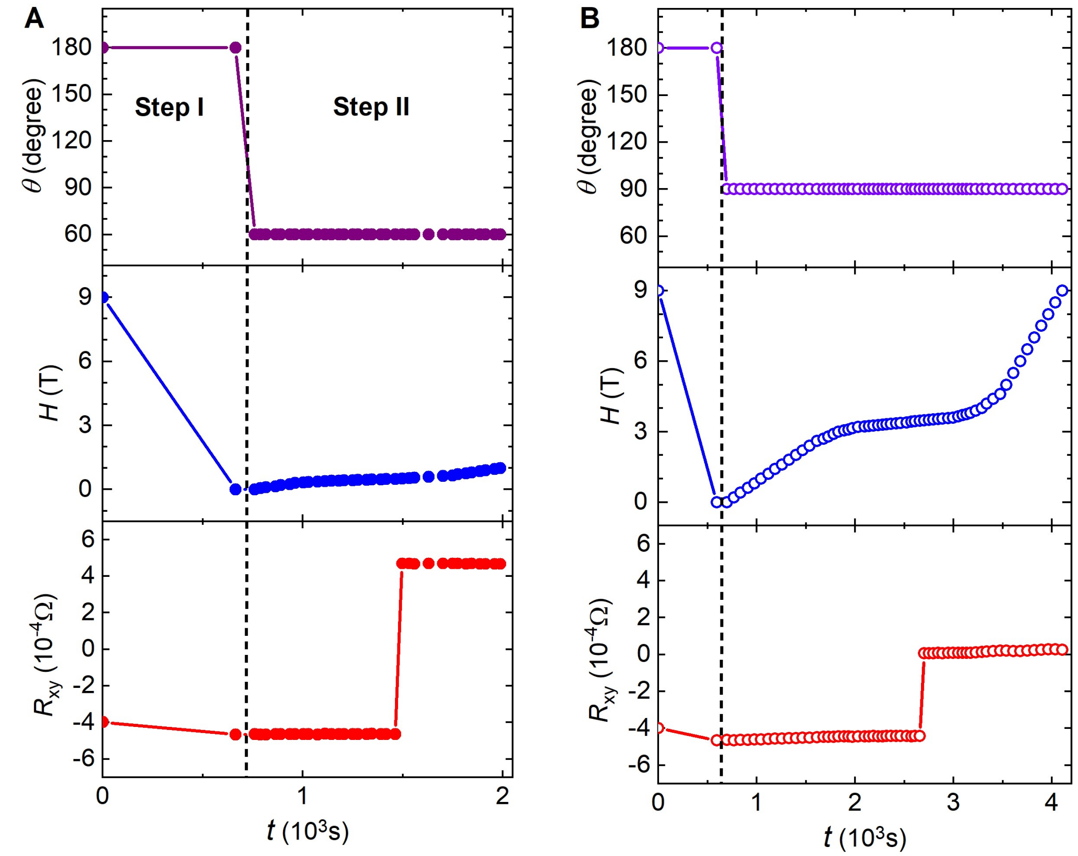

Figure S6. Procedures for preparing and driving the FM order in the R_{xy} measurements. (**A**) and (**B**) Time evolution of the angle (θ), magnetic field (H) and Hall resistance (R_{xy}) for the driving magnetic field at $\theta = 60°$ and 90°, respectively. The FM order was first prepared by sweeping the field up to $H = 9$ T along the negative direction of the c-axis, i.e., at $\theta = 180°$ and sweeping back to zero, as marked as Step I in (A). It was then driven by a magnetic field orientated at various directions, e.g., $\theta = 60°$ in (A) and $\theta = 90°$ in (B). Dense data points of R_{xy} were taken when the field sweeps up, as shown in the regime marked as Step II in (A). The slope change in the H versus time curves in Step II is due to the change of field intervals, since smaller intervals around H_s help to determine its value more accurately. The dashed lines in (A) and (B) separate Step I and Step II. The corresponding $R_{xy} \sim H$ data are plotted in Figures 1E and 1F, respectively.

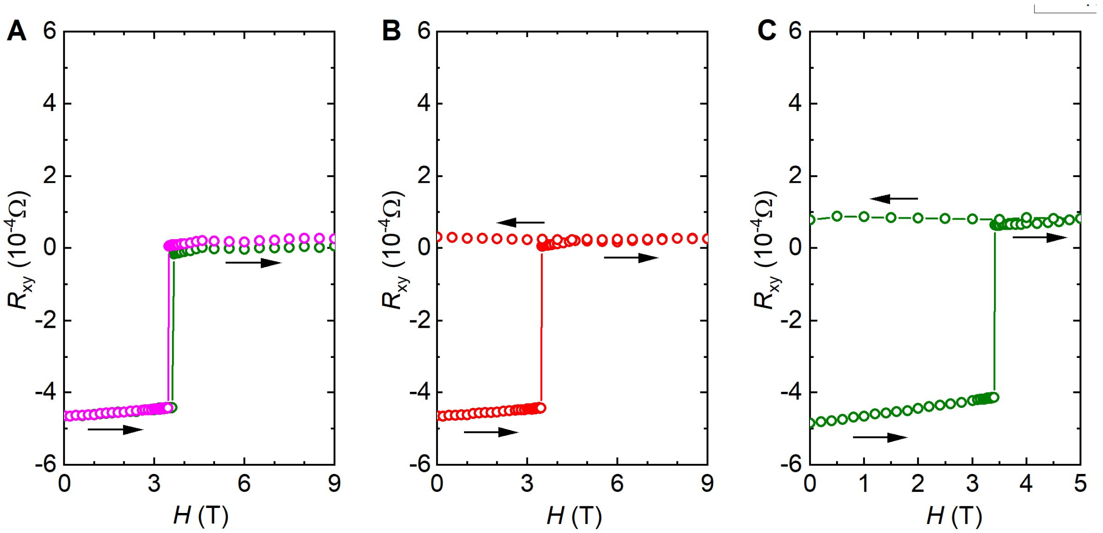

Figure S7. Repeatability of the measurements and stability of the states after the sudden change in R_{xy}. (**A**) Comparison of two sets of measurements of the same procedure (Figure S6) on Sample R1, demonstrating the repeatability. (**B**) and (**C**) Field excursion effects on the R_{xy} after the sudden change for Sample R1 up to $H = 9$ T and for Sample R2 up to $H = 5$ T and then back to zero. exhibiting the stability of the states.

Following the procedure in Figure S6, the FM order was first prepared by sweeping the field up to $H = 9$ T along the negative direction of the c-axis (i.e., at $\theta = 180°$) and sweeping back to zero. It was then driven by an in-plane field. That is, the data shown in (A)-(C) were taken by sweeping magnetic field at $\theta = 90°$. All data were taken at $T = 3$K.

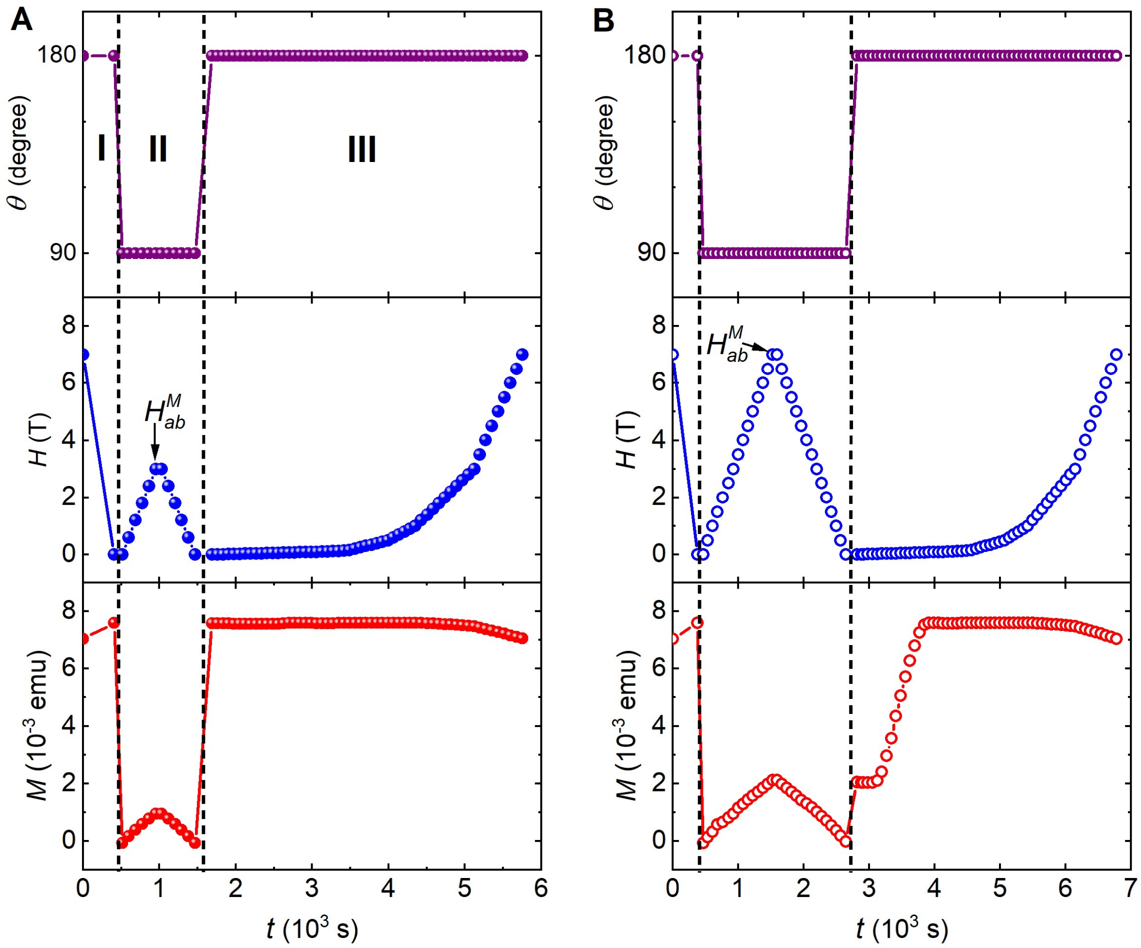

Figure S8. Magnetization measurement procedures for detecting the magnetic states of the FM structure after being driven by an in-plane field. (**A**) and (**B**) For in-plane fields up to $\boldsymbol{H_{ab}^{M}}$ = 3 T and 7 T, respectively. The FM structure was first prepared by sweeping the field up to H = 7 T along the negative direction of the c-axis, i.e., at θ = 180° and sweeping back to zero, as marked as Step I in (A). It was then driven by an in-plane magnetic field, i.e., at θ = 90°, up to various to $\boldsymbol{H_{ab}^{M}}$ and the back to zero field, as marked as Step II in (A). After that, the sample is rotated back to θ = 180° and magnetization was measured from H = 0 T to 7 T, as shown in the regime marked as Step III in (A). The change of the slope of the H versus time curves in Step III is due to the variation of the used field intervals. The dashed lines in (A) and (B) separate the three steps. The corresponding $M_{ab} \sim H$ curves (from Step II) and $M_c \sim H$ curves (from Step III) are plotted in the insets and main panels of Figures 2A and 2B, respectively.

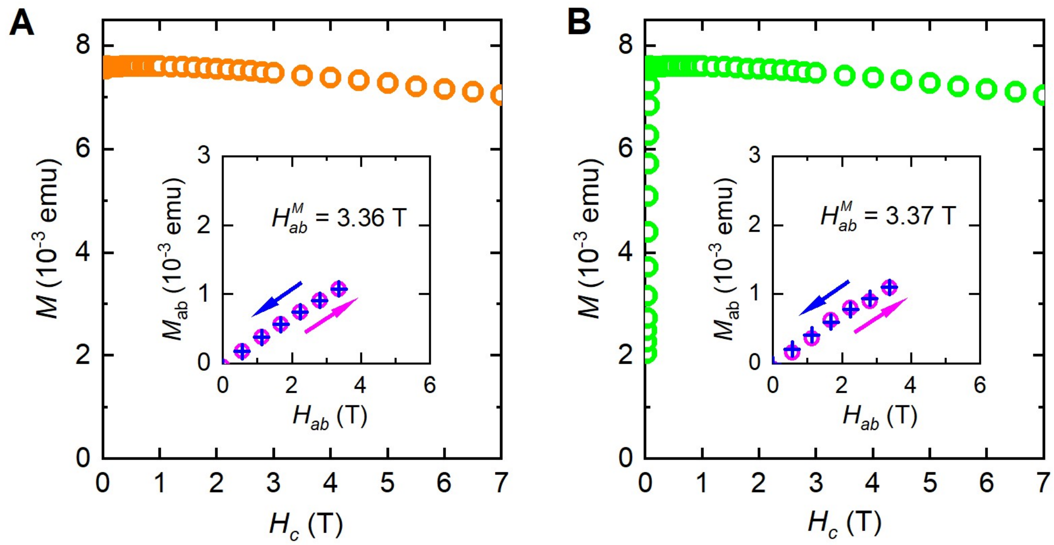

Figure S9. Magnetization of the FM order being and after driven by an in-plane field. (**A**) and (**B**) *C*-axis magnetization $\boldsymbol{M_c}$ versus magnetic field H_c curves of the FM order after driven by an in-plane field $\boldsymbol{H_{ab}}$ up to $\boldsymbol{H_{ab}^{M}}$ = 3.36 T and 3.37 T, respectively. Their insets present the in-plane magnetization $\boldsymbol{M_{ab}}$ for the FM order driven by the in-plane field $\boldsymbol{H_{ab}}$. The FM order was prepared by sweeping the field up to H = 7 T along the *c*-axis and sweeping back to zero. Detailed procedures are presented in Figure S8 and its caption. Data were taken from Sample M1 at T = 3 K.

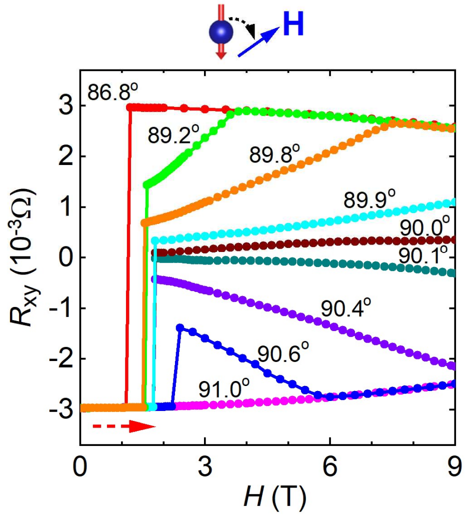

Figure S10. Field-driven FM instability at a high temperature (*T* = 80 K). The FM order with spins pointing to the negative direction of the *c*-axis was prepared by sweeping the field up to H = -9 T along the *c*-axis and sweeping back to zero. The red dashed arrow indicates the sweeping directions of the magnetic field for measuring R_{xy}. Numbers represent the angles at which the $R_{xy} \sim H$ curves were taken. The schematic on top of the main panel shows the directions of the spins in the FM order and the magnetic field as well as the definition of the angle. The magnetic field rotates in the plane perpendicular to the current direction. Data were taken from Sample R3.

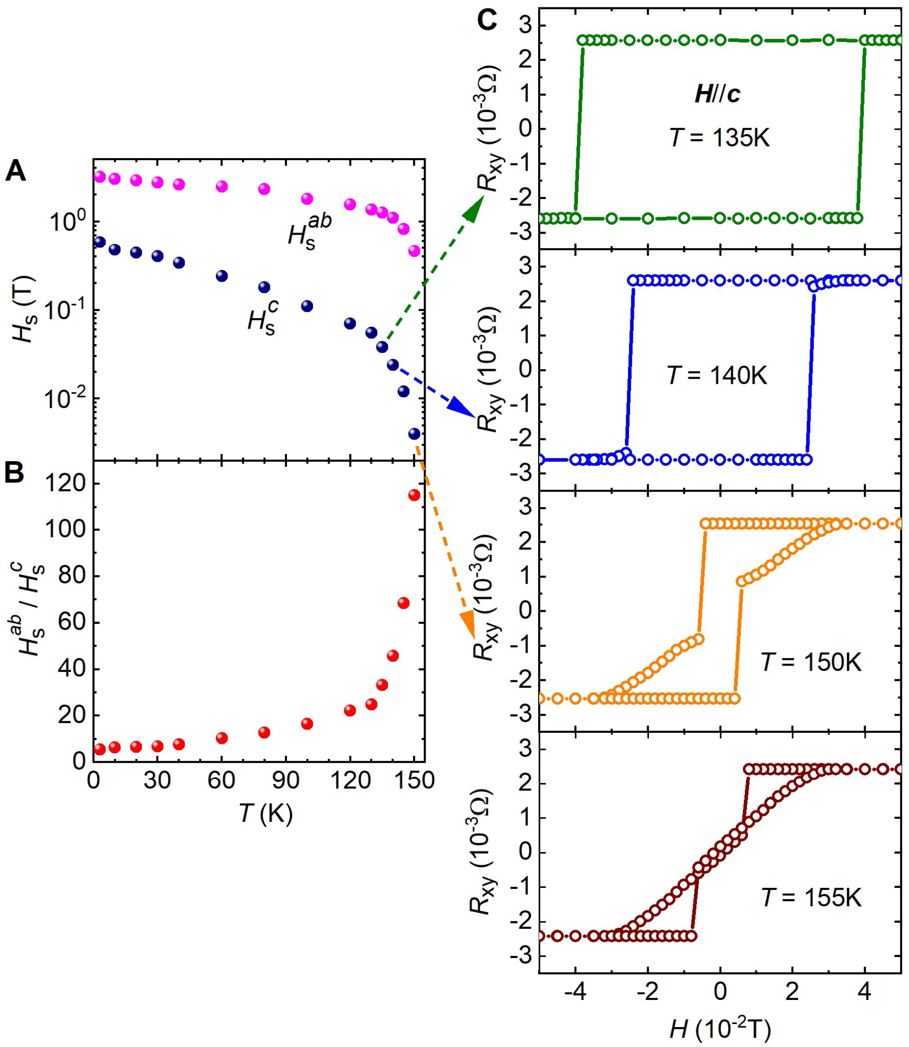

Figure S11. Temperature effects on the local FM instability and bow-tie-like hysteresis loops. (**A**) Temperature dependence of the critical fields $\boldsymbol{H_s^{ab}}$ and $\boldsymbol{H_s^{c}}$ for the local FM instability for the magnetic fields along the *ab* plane and the *c*-axis, respectively. (**B**) The temperature dependence of their ratio $\boldsymbol{H_s^{ab}/H_s^{c}}$. (**C**) $R_{xy} \sim H$ loops at high temperatures, showing bow-tie-like features. The data were taken from Sample R4.

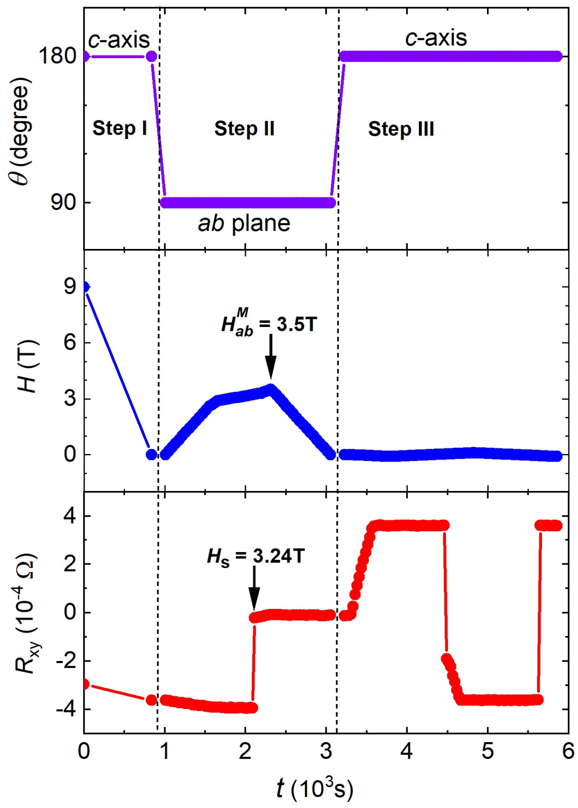

Figure S12. Transport measurement procedures for detecting the magnetic states of the FM structure after being driven by an in-plane field. The FM structure was first prepared by sweeping the field up to $H = 9$ T along the negative direction of the *c*-axis, i.e., at $\theta = 180°$ and sweeping back to zero (marked as Step I). It was then driven by an in-plane magnetic field, i.e., at $\theta = 90°$, up to various to $\boldsymbol{H_{ab}^{M}}$= 3.5 T which is larger than H_s = 3.24 T, and then back to zero field (marked as Step II). After that, the sample is rotated back to $\theta = 180°$ at which R_{xy} was measured from $H = 0$ T to $H = -0.1$ T to $H = 0.1$T and back to $H = -0.1$T, as shown in the regime marked as Step III. The $R_{xy} \sim H$ loop from $H = -0.1$ T to $H = 0.1$ T and back to $H = -0.1$ T is presented in Figure 5E. The data were taken from Sample R4 at $T = 3$ K.

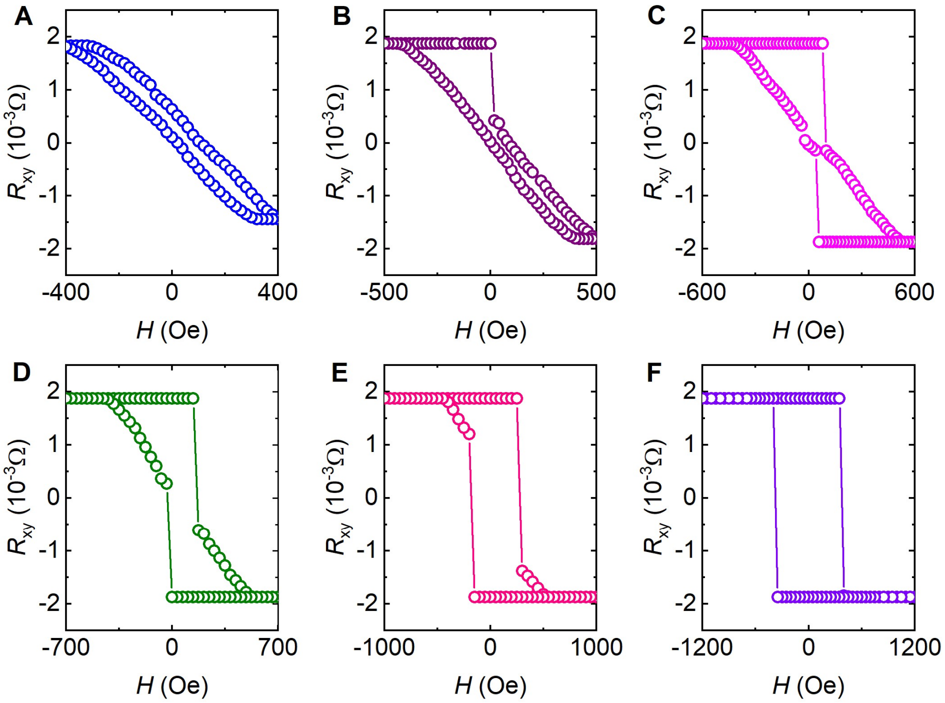

Figure S13. Bow-tie-like Hall resistance hysteresis loops. (**A**)-(**F**) Results with magnetic fields sweeping up to H = ±400 Oe, ±500 Oe, ±600 Oe, ±700 Oe, ±1 kOe and ±1.2 kOe, respectively. The $R_{xy} \sim H$ curves were taken with magnetic fields along the c-axis (θ = 180°) after the FM structure had been driven with an in-plane field up to H_{ab} = 2.2 T. The FM structure was prepared by sweeping the field up to H = -9 T along the c-axis and sweeping back to zero. Data were taken from Sample R4 at T = 100 K. Measurement procedures are the same as those for T = 3 K presented in Figure S12 and its caption.
